# Intramuscular myxoma with chondroid features: two cases expanding the morphological spectrum

**DOI:** 10.1111/his.70164

**Published:** 2026-05-30

**Authors:** Bethany Batson, Azfar Neyaz, Lucas Da Gama Lobo, Rana Naous, Stella J Lee, Ivy John

**Affiliations:** ^1^ Department of Pathology University of Pittsburgh Pittsburgh Pennsylvania USA; ^2^ Department of Radiology University of Pittsburgh Pittsburgh Pennsylvania USA; ^3^ Department of Pathology, Wexner Medical Center The Ohio State University Columbus Ohio USA; ^4^ Department of Orthopaedics Dartmouth Hitchcock Medical Center Lebanon New Hampshire USA

**Keywords:** chondroid, chondromyxoid, *GNAS* mutation, intramuscular myxoma

## Abstract

**Background:**

Intramuscular myxoma is a benign mesenchymal tumour typically composed of bland spindle to stellate cells in abundant myxoid stroma and usually characterized by GNAS mutations. Chondroid matrix has not been previously reported in intramuscular myxoma.

**Methods:**

We describe two intramuscular myxomas with chondroid‐type matrix and review the relevant radiological, histological, immunohistochemical and molecular findings, with emphasis on differential diagnostic considerations.

**Results:**

Case 1 occurred in the triceps of a 64‐year‐old woman and showed focal cartilaginous differentiation, including chondrocytes within lacunar spaces, in an otherwise classic intramuscular myxoma background. The lesional cells were negative for MUC4, S100, cytokeratin, SMA, desmin, CD34, ERG, STAT6 and EMA, and targeted sequencing detected no pathogenic alteration. Case 2 occurred in the vastus medialis of a 48‐year‐old woman and showed bland spindle to stellate cells in myxoid to collagenous stroma with foci of chondromyxoid matrix, patchy lacunar‐type spaces, and territorial basophilia. The lesional cells were positive for CD34, focally positive for SMA and negative for S100, desmin, cytokeratin and SOX10. Whole transcriptome sequencing detected no gene fusion, and targeted sequencing identified GNAS c.602G>A (p.R201H). Both tumours were deep intramuscular and markedly T2 hyperintense and showed peripheral and septal enhancement on MRI.

**Conclusions:**

These cases expand the morphological spectrum of intramuscular myxoma by documenting chondroid and chondromyxoid matrix, likely representing cartilaginous metaplasia. Awareness of this variant is important to avoid misclassification as other chondromyxoid neoplasms.

**Abbreviations**
AFCMT: acral fibrochondromyxoid tumour
EMC: extraskeletal myxoid chondrosarcoma
IHC: immunohistochemistry
IM: intramuscular myxoma
MRI: magnetic resonance imaging
STC: soft‐tissue chondroma

Intramuscular myxoma (IM) is a benign soft‐tissue tumour that is grossly well‐circumscribed yet may infiltrate between skeletal muscle fibres microscopically. These tumours most frequently occur in the proximal musculature of the upper and lower extremities, including the shoulder girdle and buttock. Histologically, IM is typically hypocellular and hypovascular, composed of bland spindle‐to‐stellate cells within abundant myxoid stroma. Hypercellular and hypervascular areas may be seen but do not alter the benign clinical course. Activating point mutations involving *GNAS* exons 8 and 9 are present in 90% of sporadic intramuscular and cellular myxomas.^1^ On magnetic resonance imaging (MRI), IMs are characteristically T2 hyperintense and T1 hypointense with thin internal septations and frequent peripheral or septal enhancement, sometimes accompanied by a perilesional fat rim.^2^ We report two IMs with chondroid or chondromyxoid differentiation and review the differential diagnostic considerations raised by this finding.

Case 1: A 64‐year‐old woman presented with a 2‐year history of a slowly growing right upper arm mass. MRI demonstrated a 3.1‐cm well‐circumscribed, lobulated mass arising within the triceps. The lesion was extremely T2 hyperintense with peripheral and septal enhancement and was favoured to represent a benign myxoid neoplasm (Figure 1A,B). Core biopsy and subsequent excision showed predominantly classic IM features, with a hypocellular, cytologically bland proliferation of spindle‐to‐stellate cells in an abundant myxoid stroma with focal cystic change. In addition, discrete foci of cartilaginous differentiation, characterized by mature chondrocytes within lacunar spaces, were identified (Figure 2A–C). Immunohistochemistry (IHC) was negative for MUC4, S100, AE1/AE3, SMA, desmin, CD34, ERG, STAT6 and EMA. Targeted next generation sequencing panel detected no pathogenic alterations.

**Figure 1:**
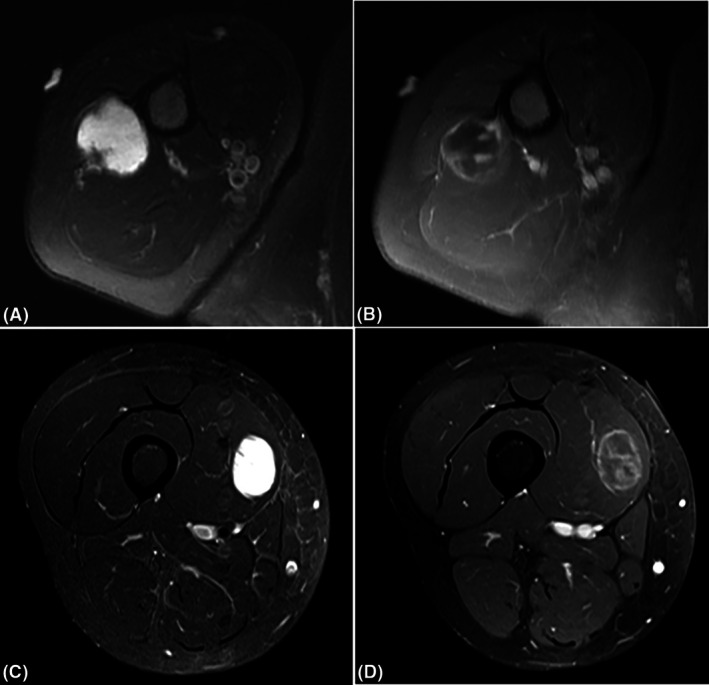
Representative radiographic images. Case 1: **(A)** Axial T2‐weighted fat‐suppressed image shows a well‐circumscribed, lobulated, extremely hyperintense intramuscular lesion involving the triceps. **(B)** Axial contrast‐enhanced T1‐weighted fat‐suppressed image demonstrates peripheral and septal enhancement. Case 2: **(C)** Axial T2‐weighted fat‐suppressed image shows a well‐circumscribed, extremely hyperintense intramuscular lesion involving the vastus medialis. **(D)** Axial contrast‐enhanced T1‐weighted fat‐suppressed image demonstrates peripheral and septal enhancement.

**Figure 2:**
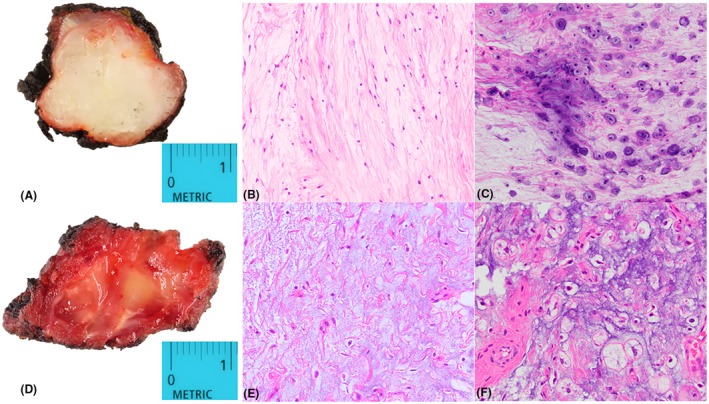
Case 1: **(A)** Gross photograph showing a gelatinous, shiny cut surface. **(B)** Representative microscopic image showing spindle‐to‐stellate cells in a myxoid background (20×). **(C)** Area of chondroid differentiation characterized by mature chondrocytes within lacunar spaces (20×). Case 2: **(D)** Gross photograph showing a gelatinous, shiny cut surface infiltrating the surrounding muscle. **(E)** Representative microscopic image showing spindle‐to‐stellate cells in a myxocollagenous background (20×). **(F)** Area of chondromyxoid matrix containing patchy lacunar‐type spaces and territorial basophilia (20×).

Case 2: A 48‐year‐old woman reported several months of fullness in the right distal medial thigh. MRI demonstrated a well‐circumscribed 5.9‐cm, intramuscular mass within the vastus medialis. The lesion was markedly T2 hyperintense and showed peripheral and septal enhancement (Figure 1C,D). Histology showed a bland spindle‐to‐stellate proliferation in a myxoid‐to‐collagenous stroma with scattered small calibre vessels. In addition, foci of chondromyxoid matrix with patchy lacunar‐type spaces and territorial basophilia were identified, merging into the myxoid background (Figure 2D–F). IHC showed CD34 positivity, focal SMA positivity and negativity for S100, desmin, AE1/AE3 and SOX10. Whole transcriptome sequencing detected no gene fusion, and a targeted NGS panel identified *GNAS* c.602G>A (p.R201H).

These two cases expand the morphological spectrum of IM by documenting chondromyxoid stroma and overt chondroid metaplasia, including areas with mature lacunar chondrocytes. This unexpected morphology raises several diagnostic considerations. The principal differential is soft‐tissue chondroma (STC), a benign extraosseous, extrasynovial cartilaginous tumour with variable myxoid change that most commonly arises in acral sites. STC is composed of lobules of well‐formed hyaline cartilage and often shows punctate or ‘grungy’ calcification. In our cases, the deep intramuscular location, absence of diffuse cartilaginous lobules or calcification, and, in Case 2, the presence of a *GNAS* p.R201H mutation favours IM with cartilaginous metaplasia over STC. Although focal myxohyaline cartilage may be present, chondroid synoviocytic neoplasm differs from IM by its predilection for the temporomandibular joint, characteristic component of large eosinophilic synoviocyte‐like cells and osteoclast‐like giant cells, and frequent *FN1* gene rearrangements.^3^ Extraskeletal myxoid chondrosarcoma (EMC) may show abundant myxoid or chondromyxoid matrix; however, these tumours are characterized by cords and clusters of uniform ovoid cells and harbour *NR4A3* rearrangements. The marked hypocellularity and lack of gene fusions in our cases favour IM over EMC. A myoepithelial tumour of soft tissue may also be considered in the differential diagnosis and is characterized by bland ovoid cells in a chondromyxoid matrix; however, these tumours typically co‐express cytokeratin and/or EMA with S100 and often harbour *EWSR1* gene fusions. Both tumours in this series were negative for cytokeratin, EMA and S100 and were fusion‐negative (Case 2). Ossifying fibromyxoid tumour can rarely show cartilaginous metaplasia but often exhibits a peripheral ossified shell, variable S100/desmin expression and *PHF1*‐related rearrangements, none of which are present in IM.^4^ Finally, acral fibrochondromyxoid tumour (AFCMT) is characterized by chondrocyte‐like tumour cells set within abundant chondromyxoid stroma. However, AFCMT is restricted to the digits and typically shows ERG and CD34 positivity and harbours a recurrent *THBS1::ADGRF5* fusion. The deep, intramuscular location and absence of this fusion exclude that diagnosis.^5^

Ectopic chondrogenesis in soft tissue can be driven by microenvironmental cues that activate hypoxia‐responsive and *TGF‐β/BMP* pathways, often reinforced by mechanical loading. In vitro and in vivo studies show that hypoxia induces *HIF‐1α*‐dependent *SOX9* activity and promotes chondrocyte gene expression, while mechanical compression enhances chondrogenic differentiation and matrix deposition.^6^, ^7^, ^8^ The markedly myxoid and relatively hypovascular matrix of IM provides a plausible microenvironment for such differentiation. This notion is further supported by rare reports of chondroid metaplasia in cardiac myxoma.^9^, ^10^ It is important to note, however, that cardiac and IMs are clinicopathologically and genetically distinct. Cardiac myxomas frequently show *PRKAR1A* inactivation and, in contrast, lack *GNAS* mutations.^11^

In summary, these two cases demonstrate that IMs can harbour areas of chondroid or chondromyxoid matrix, most plausibly through cartilaginous metaplasia within a myxoid, hypovascular microenvironment. Awareness of this morphological variant is essential to prevent misclassification. Careful correlation with imaging findings, recognition of otherwise classic background IM morphology, and judicious use of IHC and *GNAS* mutation testing allow for confident distinction from its mimics.

## Author contributions

BB: Data acquisition and writing original manuscript draft. AZ, RN and SJL: Manuscript review and editing. LDGL: Radiologic data acquisition and manuscript review and editing. IJ: Study design, supervision and manuscript review and editing. All authors read and approved the final manuscript.

## Funding information

This work was supported in part by an internal grant from the UPMC/University of Pittsburgh Department of Pathology.

## Conflicts of Interest

The authors declare no competing interests.

## Consent

Informed consent for publication was obtained.

## Data Availability

Data sharing is not applicable to this article as no datasets were generated or analysed during the current study.
